# Utility of standard diffusion-weighted magnetic resonance imaging for the identification of ischemic optic neuropathy in giant cell arteritis

**DOI:** 10.1038/s41598-022-20916-y

**Published:** 2022-10-03

**Authors:** L. A. Danyel, M. Miszczuk, C. Pietrock, B. T. Büge, K. Villringer, G. Bohner, E. Siebert

**Affiliations:** 1 Department of Neurology, Charité – Universitätsmedizin Berlin, Corporate Member of Freie Universität Berlin and Humboldt Universität zu Berlin, Berlin, Germany; 2 Institute of Neuroradiology, Charité – Universitätsmedizin Berlin, Corporate Member of Freie Universität Berlin and Humboldt Universität zu Berlin, Berlin, Germany; 3 Center for Stroke Research Berlin, Charité – Universitätsmedizin Berlin, Corporate Member of Freie Universität Berlin and Humboldt Universität zu Berlin, Berlin, Germany

**Keywords:** Vasculitis syndromes, Neurovascular disorders

## Abstract

This study assessed diffusion abnormalities of the optic nerve (ON) in giant cell arteritis (GCA) patients with acute onset of visual impairment (VI) using diffusion-weighted magnetic resonance imaging (DWI). DWI scans of GCA patients with acute VI were evaluated in a case-control study. Two blinded neuroradiologists assessed randomized DWI scans of GCA and controls for ON restricted diffusion. Statistical quality criteria and inter-rater reliability (IRR) were calculated. DWI findings were compared to ophthalmological assessments. 35 GCA patients (76.2 ± 6.4 years; 37 scans) and 35 controls (75.7 ± 7.6 years; 38 scans) were included. ON restricted diffusion was detected in 81.1% (Reader 1) of GCA scans. Localization of ON restricted diffusion was at the optic nerve head in 80.6%, intraorbital in 11.1% and affecting both segments in 8.3%. DWI discerned affected from unaffected ON with a sensitivity, specificity, positive and negative predictive value of 87%/99%/96%/96%. IRR for ON restricted diffusion was κ_inter_ = 0.72 (95% CI 0.59–0.86). DWI findings challenged ophthalmologic diagnoses in 4 cases (11.4%). DWI visualizes anterior and posterior ON ischemia in GCA patients with high sensitivity and specificity, as well as substantial IRR. DWI may complement the ophthalmological assessment in patients with acute VI.

## Introduction

Giant cell arteritis (GCA) is a chronic granulomatous vasculitis of middle- to large-sized arteries with frequent involvement of the cranial vasculature. It is considered a disease of the elderly; typically affecting people of 50 years and older and further increasing in prevalence with age^1^. An US study estimates the lifetime risk of developing GCA at 0.5% for men and 1% for women^2^. Due to its proclivity to affect the ophthalmic and posterior ciliary arteries, GCA patients may develop a wide range of ocular complications^3^. Among them, arteritic ischemic optic neuropathy (ION, anterior or posterior) may cause severe and persistent visual impairment (VI) in the affected eye. Hence, GCA is considered a neuro-ophthalmological emergency that, if suspected, requires fast diagnostic workup and early corticosteroid treatment^4^. In a clinical setting however, identifying a possible GCA complication in a patient presenting with new onset of VI may be challenging if ophthalmological assessment is hindered by restricted vision of the eye fundus or if concurrent age-related ocular disorders are misinterpreted as causative—especially if other clinical signs of GCA are not apparent^5^. Posterior ION represents a particular diagnostic challenge as ophthalmoscopic examination during the acute phase reveals no abnormalities, but optic disc pallor/atrophy usually develops after 6–8 weeks^6^.

Diffusion-weighted magnetic resonance imaging (DWI-MRI) is a well-established imaging technique that allows for the non-invasive detection of cerebral ischemia through measurement of the apparent diffusion coefficient (ADC) of water within the brain tissue^7^. Furthermore, numerous case reports have indicated the presence of DWI-related abnormalities of the optic nerve in non-arteritic ION of variable etiology^8–25^. In a case-control study, Bender et al. evaluated DWI scans of patients with acute visual deficit. Here, DWI identified all cases of ION through restricted diffusion of the optic nerve^26^.

Together, these findings indicate that standard DWI-MRI may prove useful for the identification of ischemic neuro-ophthalmologic complications in GCA. Notably, two case reports describe DWI-related abnormalities of the optic nerve in GCA patients presenting with new onset of vision loss^27,28^. Based on our review of the MEDLINE database, we conclude that no systematic studies assessing the clinical utility of standard DWI in GCA-related vision-loss exist as of today. Hence, the goal of our study was to characterize optic nerve DWI abnormalities in GCA patients with acute onset of severe VI compared with their respective ophthalmological findings.

## Materials and methods

This retrospective case–control study evaluated DWI abnormalities of the optic nerve in GCA patients presenting with acute onset of persisting VI, treated at our institution between January 2015 and December 2021. Ethical approval was obtained from the local ethics committee (Charité Universitätsmedizin Berlin, approval number EA1/177/19). All methods were performed in accordance with relevant guidelines/ regulations. Written informed consent was obtained from all participants.

### Patient selection

First, a medical database inquiry was performed to identify potential GCA patients with available MRI based on codes of the *International Classification of Diseases* (ICD; *M31.5* and *M31.6*) and the German *Operation and Procedure Classification System* (OPS; *3–800* and *3–820*). Data records of hereby-identified candidates were screened for information relevant to GCA diagnostic criteria: GCA was diagnosed if a patient met 3 of the 5 1990 American College of Rheumatology criteria for the classification of GCA^29^ or fulfilled entry criteria of the Giant-Cell Arteritis Actemra (*GiACTA*) trial^30^. As distinguished from *GiACTA*, the presence of a sonographic “halo” sign (homogeneous, hypoechoic wall thickening of the superficial temporal artery^31^) was considered as evidence of large-vessel vasculitis.

GCA patients were included in the study if they (1) presented with new onset of monocular or binocular VI and corresponding visual acuity (VA) of worse than 0.33 (> 0.48 LogMAR; moderate VI or worse according to the WHO International Statistical Classification of Diseases and related Health Problems 10th revision, 2016) and (2) had cranial DWI performed within 30 days after clinical onset of VI. Patients without available ophthalmologic assessments were not considered for the study.

Patient medical records were reviewed in consideration of demographic data, medical history, clinical presentation of GCA, ophthalmological consultations and laboratory findings. If available, results of temporal artery biopsy (TAB) and superficial temporal artery (STA) ultrasound were assessed. In addition, records of computed tomography and magnetic resonance angiography were screened for evidence of large-vessel vasculitis.

Lastly, age- and sex-matched controls were randomly selected from a cohort of transient ischemic attack and ischemic stroke patients, treated in our department between July 2015 and February 2021. All control patients had cerebral DWI-MRI performed utilizing comparable technical protocols.

### Diffusion weighted MR imaging analysis

DWI-MRI was performed on a 3 T scanner (Skyra, Siemens, Erlangen, Germany) and two 1.5 T scanners (Aera, Siemens, Erlangen, Germany), each with a 20-channel head coil. Another 3 T scanner (Trio, Siemens, Erlangen, Germany) with a 32-channel head coil was used to acquire diffusion tensor imaging (DTI) sequences used for DWI calculation. Trace DWI b = 1000 s/mm2 images (EPI-DWI sequences or calculated from EPI-DTI sequences) with field strengths of either 1.5 or 3 T and slice thicknesses of either 2.5 mm or 3 mm were evaluated. Time span between onset of VI and DWI was noted and classified into four subgroups: (1) ≤ 72 h, (2) > 72 h–7 d, (3) > 7 d–14 d, (4) > 14–30d.

A board-certified neuroradiologist (Reader 1; > 15 years of experience in MR imaging), and a radiology resident in training for neuroradiology (Reader 2; 2 years of neuroradiological experience) were independently presented a mixed set of imaging studies of GCA cases and controls for image analysis. Both readers were blinded for patient diagnosis and side of VI. Images were evaluated for presence of optic nerve restricted diffusion, indicated by local signal increase in the evaluable parts of the optic nerve (optic nerve head, intraorbital, intracanalicular or intracranial portion). Corresponding ADC map images were evaluated for visually correlating low signal. Finally, Reader 2 additionally noted the presence of cerebral restricted diffusion.

### Statistical analysis

Statistical analyses were performed with IBM SPSS Statistics software (IBM SPSS Statistics for Windows, Version 25.0. Armonk, NY: IBM Corp.). Sensitivity, specificity, and positive and negative predictive values of DWI for the identification of GCA patients were calculated for each reader. Interrater agreement was analyzed through unweighted Cohen’s κ, which was calculated through the observed Pr(a) and expected percentage of agreement Pr(e)$:\upkappa = \frac{\mathrm{Pr}\left(a\right) - \mathrm{Pr}(e)}{(1- \mathrm{Pr}\left(e\right))}$. The interpretation of agreement for kappa was categorized as: poor (κ < 0.00); slight (0.00 ≤ κ ≤ 0.20), fair (0.21 ≤ κ ≤ 0.40), moderate (0.41 ≤ κ ≤ 0.60), substantial (0.61 ≤ κ ≤ 0.80) or almost perfect (κ > 0.80), respectively. Qualitative variables are presented as number (percentage) and quantitative variables as mean ± SD.

## Results

### Patient data and clinical characteristics

**Figure 1:**
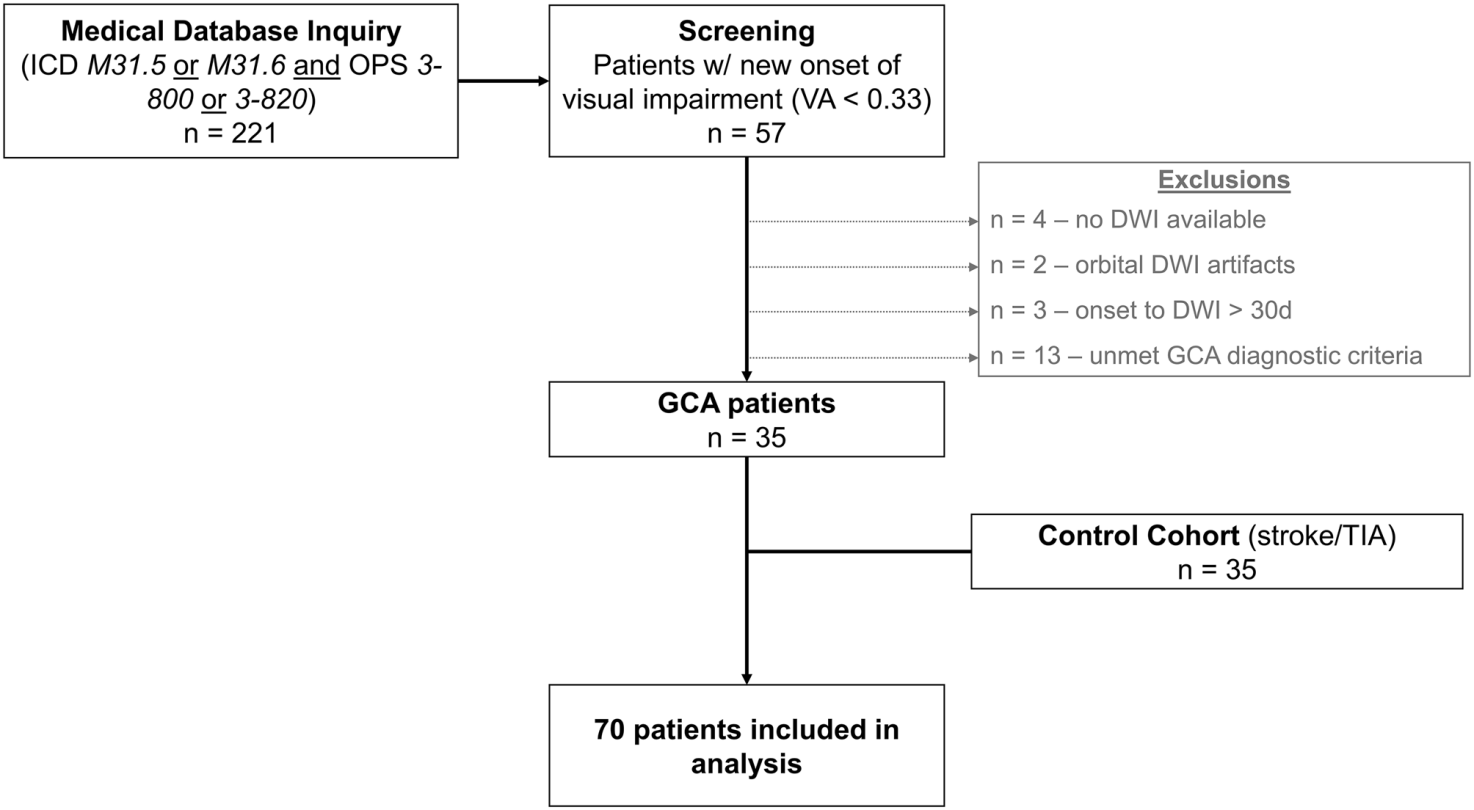
Flow diagram of the patient selection process. Abbreviations: DWI, diffusion-weighted imaging; GCA, giant cell arteritis; ICD, International Classification of Diseases; OPS, Operation and Procedure Classification System; TIA, transient ischemic attack; VA, visual acuity.

Our medical database inquiry identified 221 potential cases of GCA with available MRI scans. Of those, 57 patients presented with new onset of VI and a corresponding visual acuity < 0.33. 9 patients were excluded due to missing DWI sequences, the presence of severe orbital DWI artifacts or a > 30 d time interval between clinical onset of VI and DWI. Another 13 patients did not meet our GCA diagnostic criteria and thus were not considered for analysis. In total, 35 GCA patients with new onset of severe VI (76.2 ± 6.4 years; 9 [25.7%] male, 26 [74.3%] female) and 35 age- and sex-matched controls (ischemic stroke or transient ischemic attack; 75.7 ± 7.6 years; 9 [25.7%] male, 26 [74.3%] female) were included in the study. Figure *1* illustrates the patient selection process.

Table 1 details clinical characteristics and laboratory findings of GCA patients. GCA patient diagnostic criteria, TAB, STA-ultrasonography and angiography results are presented in Table 2. New localized headache was reported in 27 (77.1%), scalp and/or STA tenderness in 8 (22.9%), decreased pulsation of STA in 10 (28.6%) and mouth and/or jaw pain on mastication in 14 (40.0%) patients. 85.7% of GCA patients had a history of ESR ≥ 50 mm/h. TAB was performed in 20 patients (57.1%) with confirmation of GCA in 13/20 cases (65.0%). Inflammatory infiltration of the superficial temporal artery wall (hypoechoic vessel wall thickening “halo sign”) was identified by ultrasonography in 22/34 (64.7%) patients.

**Table 1:** Clinical and laboratory characteristics of GCA patients and radiological features of DWI-MRI.

	GCA patients	Control cohort
	n = 35	n = 35
Age (mean ± SD)	76.2 ± 6.4 years	75.7 ± 7.6 years
Female (n, %)	26 (74.3%)	26 (74.3%)
**GCA symptoms**		
New localized headache (n, %)	27 (77.1%)	
Scalp/STA tenderness (n, %)	8 (22.9%)	
STA decreased pulsation (n, %)	10 (28.6%)	
Mouth/jaw pain on mastication (n, %)	14 (40.0%)	
**Laboratory findings**		
History of ESR ≥ 50 mm/h (n, %)	30 (85.7%)	
ESR (mm/h; mean ± SD)	62.8 ± 15.5	
C-reactive protein (mg/l; mean ± SD)	77.6 ± 67.9	
Hemoglobin (g/dl; mean ± SD)	12.1 ± 1.3	
Platelet count (/nl; mean ± SD)	408.3 ± 96.7	
WBC count (/nl; mean ± SD)	10.6 ± 3.6	
**MRI parameters**		
Scans	n = 37	n = 38
Field strength		
1.5 T (n, %)	17 (46.0%)	21 (55.3%)
3 T (n, %)	20 (54.1%)	17 (44.7%)
Slice thickness		
2.5 mm (n, %)	10 (27.0%)	11 (29.0%)
3 mm (n, %)	27 (73.0%)	27 (71.1%)
**Sequence type**		
DWI-EPI (n, %)	30 (81.1%)	29 (76.3%)
DTI (n, %)	5 (13.5%)	9 (23.7%)
RESOLVE (n, %)	1 (2.7%)	–
RESOLVE DTI (n, %)	1 (2.7%)	–
**Acute brain infarction (n, %)**	6 (16.2%)	7 (18.4%)

*DWI* diffusion-weighted imaging, *DTI* diffusion tensor imaging, *ESR* erythrocyte sedimentation rate, *GCA* giant cell arteritis, *LVV* large vessel vasculitis, *MRI* magnetic resonance imaging, *STA* superficial temporal artery, *TAB* temporal artery biopsy, *US* ultrasound, *WBC* white blood cell count.

**Table 2:** GCA patient cohort: diagnostic criteria, TAB, STA ultrasonography and angiography results.

	GCA patients
	(n = 35)
**GCA diagnostic criteria**	
ACR criteria met (n, %)	30 (85.7%)
Adj. GIACTA trial criteria met (n, %)	29 (82.9%)
ACR *and* adj. GIACTA trial criteria met (n, %)	24 (68.6%)
ACR *or* adj. GIACTA trial criteria met (n, %)	35 (100%)
**Evidence of vasculitis**	
STA US	
US performed (n, %)	34 (97.1%)
STA Halo (n, %)	22/34 (64.7%)
Angiographic LVV	
Angiography available	29 (82.9%)
Presence of LVV	12/29 (41.4%)
**Temporal artery biopsy**	
TAB performed (n, %)	20 (57.1%)
TAB specimen length (mm; mean ± SD)	16.1 ± 10.1
GCA-positive TAB (n, %)	13/20 (65.0%)
Patients w/ STA Halo *or* angiographic LVV *or* GCA-positive TAB (n, %)	30 (85.7%)
Patients w/ STA Halo *and* angiographic LVV (n, %)	7 (20.0%)
Patients w/ STA Halo *and* GCA-positive TAB (n, %)	8 (22.9%)
Patients w/ GCA-positive TAB *and* angiographic LVV (n, %)	4 (11.4%)
Patients w/ STA Halo *and* angiographic LVV *and* GCA-positive TAB (n, %)	2 (5.7%)

*ACR* american college of rheumatology, *adj* adjusted, *GiACTA trial* giant-cell arteritis actemra trial, *GCA* giant cell arteritis, *LVV* large vessel vasculitis, *STA* superficial temporal artery, *TAB* temporal artery biopsy, *US* ultrasound.

VI was right-sided in 12 (34.3%), left-sided in 17 (48.6%) and bilateral in 6 (17.1%) cases (at the time of discharge). A total of 41 eyes were affected. Notably, 3 patients (8.6%) who initially presented with unilateral loss of vision developed additional VI of the contralateral eye during hospitalization.

Main causes of VI as determined by ophthalmological examination were as follows: anterior ischemic optic neuropathy in 28 (68.3%), central retinal artery occlusion in 6 (14.6%) and branch retinal arteriolar occlusion (BRAO) in 2 eyes (4.9%). Other diagnoses were: optic nerve atrophy, combined AION and retinal ischemia (unspecified), combined BRAO and retinal/subretinal bleeding, ocular ischemic syndrome and age-related macular degeneration (each in 1 eye [2.4%], respectively).

### Diffusion weighted MR imaging analysis

A total of 75 MRI scans (37 in GCA patients, 38 in controls) were evaluated. Table 1 details technical data such as field strengths, slice thicknesses and sequence types employed. Time-to-DWI (from onset of VI) in GCA patients was as follows: ≤ 72 h for 12 (32.4%), > 72 h–7 d for 14 (37.8%), > 7 d–14 d for 6 (16.2%) and > 14 d–30 d for 5 (13.5%) scans.

Optic nerve restricted diffusion in GCA patients was detected in 30 of 37 scans (81.1%) or 36 of 74 eyes examined (48.6%) with visually correlating low ADC map signal in 83.3% (Reader 1) and 24 of 37 scans (64.9%) or 27 of 74 eyes (36.5%) with visually correlating low ADC map signal in 96.3% (Reader 2), respectively. Reader 2 falsely attributed optic nerve diffusion restriction to one eye in a DWI scan of a control patient. Localization of ON restricted diffusion was as follows (Reader 1): optic nerve head in 29 eyes (80.6%), intraorbital in 4 (11.1%) and affecting both segments in 3 eyes (8.3%). No restricted diffusion was found in the intracanalicular or intracranial portion of the ON. Finally, in 6 patients (17.1%) neither of the readers detected DWI abnormalities of the optic nerve (2 patients with AION, 2 with BRAO, 1 with CRAO and 1 with optic nerve atrophy as per ophthalmological report).

**Figure 2:**
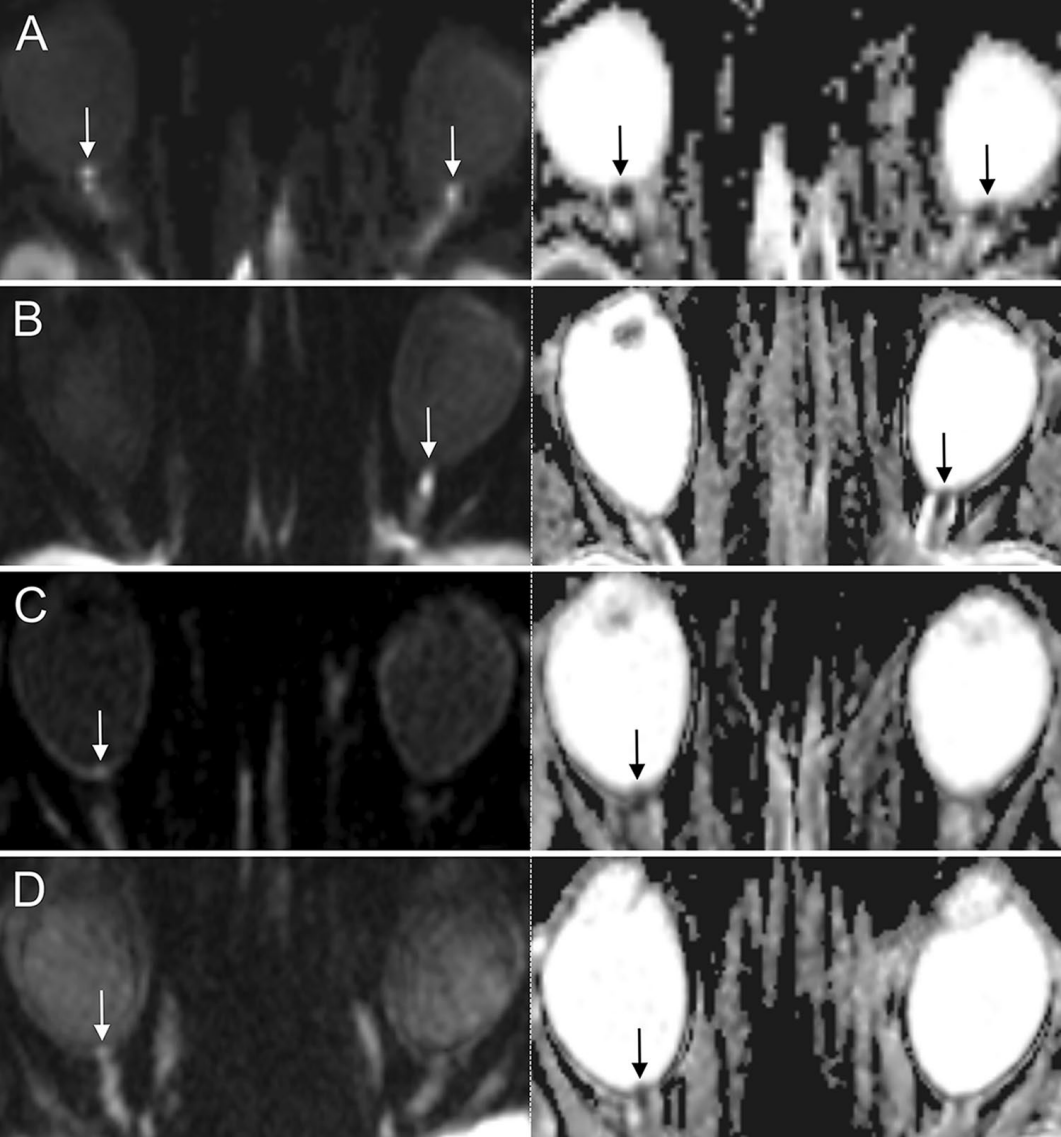
Examples of 3 T DWI-MRI in 4 GCA patients presenting with acute arteritic AION (left row: DWI b = 1000 s/mm2, right row: corresponding ADC images). (**A**, **B**): Distinct findings of ONH restricted diffusion in 2 patients with AION (white arrows; bilateral (**A**) and left-sided (**B**)). Note the marked qualitative ADC reduction in both cases (black arrows). (**C**, **D**) Subtle findings of ONH restricted diffusion (white arrows) and corresponding visually qualitative ADC reduction (black arrows) in 2 patients with right-sided AION. Abbreviations: AION, anterior ischemic optic neuropathy; DWI-MRI, diffusion-weighted magnetic resonance imaging; GCA, giant cell arteritis; ONH, optic nerve head.

**Figure 3:**
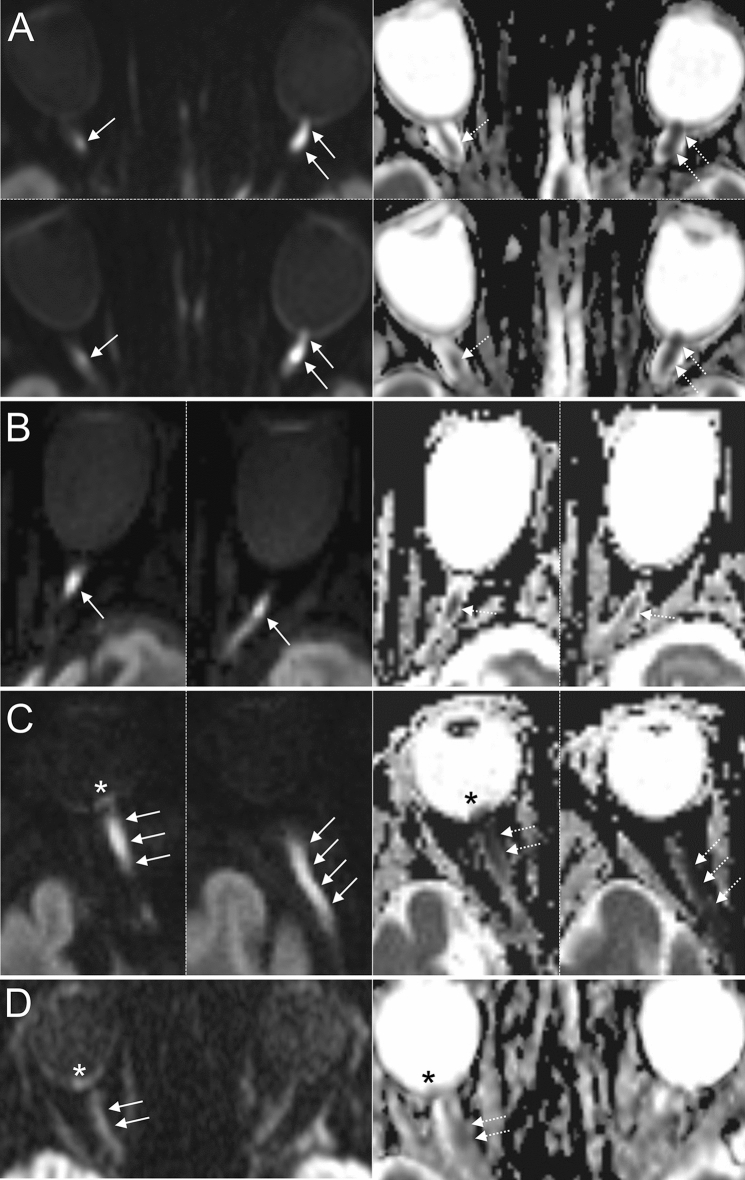
Examples of DWI-MRI in 4 patients with GCA and arteritic ION involving the intraorbital part of the ON (left row: DWI b = 1000 s/mm2, right row: corresponding ADC images). (**A**) Two consecutive DWI-MRI slices of a GCA patient showing bilateral restricted diffusion of the ON (white arrows; right: intraorbital, left: bordering ONH and intraorbital portion) and corresponding ADC reduction (dotted arrows) suggestive of right posterior ION and left anterior ION. (**B**) Restricted diffusion of the intraorbital part of the ON on two consecutive DWI-MRI slices (white arrows) and corresponding ADC reduction (dotted arrows) in a patient with left ION. (**C**) Acute ocular ischemic syndrome in GCA: Two consecutive DWI-MRI slices showing extensive restricted diffusion of the ON involving the ONH and the intraorbital portion (white arrows) with corresponding qualitative ADC reduction (dotted arrows). Note the presence of retinal diffusion restriction indicating concomitant retinal ischemia (asterisks). The findings are indicative of combined arteritic anterior/posterior ION and retinal artery occlusion. (**D**) Example of 1.5 T DWI showing right-sided restricted diffusion of the intraorbital optic nerve (white arrows) and retina (white asterisk) with subtle qualitative ADC reduction. Abbreviations: ION, ischemic optic neuropathy; DWI-MRI, diffusion-weighted magnetic resonance imaging; GCA, giant cell arteritis; ON, optic nerve; ONH, optic nerve head.

Overall sensitivity, specificity, positive and negative predictive value of DWI for the identification of GCA patients were 81%/100%/100%/84% (Reader 1) and 65%/97%/96%/74% (Reader 2). In GCA patients with ophthalmological diagnosis of ischemic optic neuropathy and controls, DWI discerned affected from unaffected optic nerves with a sensitivity, specificity, positive and negative predictive value of 87%/99%/96%/96% (Reader 1) and 70%/98%/91%/91% (Reader 2). Interrater reliability for optic nerve restricted diffusion was “substantial” with κ_inter_ = 0.72 (95% CI 0.59–0.86). Figure 2 illustrates representative examples of optic nerve head restricted diffusion in GCA patients. Cases of intraorbital restricted diffusion of the optic nerve are shown in Fig. 3.

Finally, in 4 (11.4%) patients, ophthalmological diagnosis was challenged or complemented by DWI findings (concordant rating of both readers): The first patient presented with right-sided loss of vision, incomplete external ophthalmoplegia, edema of the corneal stroma and ocular hypotony. Extensive scalp necrosis was noted. An ophthalmoscopic examination was not possible due to restricted vision of the eye fundus, but swelling of the optic nerve papillae was noted in orbital sonography. The patient was diagnosed with ocular ischemic syndrome. Assessment of DWI revealed restricted diffusion of the retina, as well as the optic nerve (optic nerve head and intraorbital portion) of the right eye indicative of retinal ischemia and combined AION/PION (Fig. 3C). The second patient noted new VI of the right eye upon waking up in the morning. Ophthalmological examination identified concurrent right-sided abducens nerve palsy. New VI was initially ascribed to age-related macular degeneration. DWI however, revealed restricted diffusion of the optic nerve bordering the optic nerve head and intraorbital portion suggestive of AION. Notably, ophthalmological follow-up examination after 3 months revealed right-sided optic nerve head pallor/atrophy. The third patient was admitted to our neurovascular department with a diagnosis of central retinal artery occlusion of the left eye. However, left-sided restricted diffusion of the intraorbital portion of the optic nerve was revealed through DWI suggesting PION. Atrophy of the optic nerve was noted in an optical coherence tomography examination 6-months later. Finally, the fourth patient presented with vision loss of the right eye with fundoscopic examination revealing retinal edema with cherry red spot sign indicative of central retinal artery occlusion. Although retinal restricted diffusion of the right eye was in accordance with a diagnosis of CRAO^32,33^, DWI further revealed right-sided optic nerve restricted diffusion of the intraorbital portion suggesting concurrent PION (Fig. 3D).

## Discussion

In recent years, a growing amount of scientific literature has strengthened the role of imaging in large vessel vasculitides with increasing clinical significance of MRI for the diagnosis and management of patients with GCA^34^. Contrast-enhanced, high-resolution MRI (CME-MRI) has emerged as an alternative modality to assess arteritic involvement of the superficial temporal and occipital arteries, while TAB is still recommended to secure diagnosis, especially if GCA cannot be confirmed or excluded based on clinical features, laboratory results and/or imaging^35^.

CME-MRI may help to detect inflammation of the intracranial arteries^36^. Affected vessels are typically identified by wall thickening and mural contrast enhancement. Interestingly, numerous case reports^37–44^ and systematic investigations^45–47^ have described arteritic involvement of the ophthalmic and posterior ciliary arteries, as well as perineural enhancement (PNE) of the optic nerve in GCA patients with ION. However, the studies available suggest that the aforementioned imaging features can be observed in eyes of GCA patients unaffected by vision loss^38,43,45–47^. Cerebral DWI has long been an established method to identify ischemic stroke, which is a feared complication in GCA patients with cranial artery involvement^48^. However, a growing number of case reports^8–25,49^ and systematic studies^26,32,33,50,51^ substantiate the presence of DWI abnormalities in non-arteritic ischemic lesions of the optic nerve and retina. Additionally, DTI studies by Fang and Wang et al. have observed changes in ON fractional anisotropy in subacute AION of unspecified etiology^52,53^. However, only a few investigations have focused on the utility of routine MRI DWI in GCA patients with acute VI^27,28^. Here we present the imaging findings of the largest cohort of GCA patients with acute onset of VI examined by standard DWI to date.

Our data substantiate that arteritic AION is the most frequent cause of severe VI among GCA patients, which is well in accordance with past clinical studies on ocular complications in GCA^54^. In GCA patients with ophthalmological diagnosis of ION, standard DWI-MRI at 1.5 T or 3 T was capable of identifying the optic nerves affected by ischemia with good diagnostic accuracy and “substantial” interrater reliability. DWI of AION patients typically featured dot-shaped restricted-diffusion of the optic nerve head (Fig. 2), resembling the “central bright spot sign” Remond et al. describe in their 2017 study utilizing contrast-enhanced, flow-compensated, 3D T1-weighted MRI^55^. ONH ischemia in arteritic AION is typically caused by thrombotic occlusion of the posterior ciliary arteries, which are predominantly affected in GCA^56^.

In contrast, ischemia of the posterior intraorbital part of the optic nerve in arteritic PION results from direct arteritic involvement of the ophthalmic artery or its orbital branches^56^. PION has historically been described as a diagnosis of exclusion^57^, characterized by normal optic disc and fundus on initial ophthalmoscopic examination, but progressive development of optic disc pallor/atrophy within 6–8 weeks. PION may occur as a perioperative complication (surgical PION), but in patients aged older than 50 years without prior surgery all cases of PION are highly suggestive of GCA^56^. Diagnosis of PION may pose a challenge to the ophthalmologist, especially in the presence of concurrent ocular disease^5^.

Notably, none of our GCA patients had an ophthalmological diagnosis of PION. However, restricted diffusion of the intraorbital portion of the optic nerve was concordantly identified by both readers in 5 patients. In 2 cases, ophthalmologic evaluation (presence of optic disc edema) determined AION as diagnosis at discharge, which suggest that optic nerve edema extended from the intraorbital portion to the optic nerve head. In the remaining 3 cases, diagnosis of PION could not be established due to concurrent ocular disease. The results of our systematic investigation support the notion that DWI-MRI is able to visualize optic nerve ischemia in acute PION, as indicated by previous case reports^8,10,12,13,16–19,25^. This finding is of major significance as DWI-MRI allows for an early diagnosis of clinically suspected PION in the *acute phase* of the disease, thereby possibly reducing the time delay to the initiation of corticosteroid treatment in GCA.

Not all cases of ischemic optic neuropathy were identified by DWI-MRI: Reader 1 failed to identify 4, while reader 2 missed 9 optic nerves affected by AION. We hypothesize that this variability of false negative ratings are mainly attributed to the difference in neuroradiological training (> 15 years vs. 2 years of neuroradiological experience), as optic nerve head restricted diffusion in AION may present as a rather subtle finding on DWI-MRI (Fig. 2). However, 3 eyes affected by AION were falsely rated negative by both readers. All of the three consistently false-negative rated patients were examined using 1.5 T DWI-MRI, which may be insufficient to induce a perceivable signal increase in the ONH affected by mild AION. DWI-MRI protocols with increased field strength and finer slice thickness may help to improve visibility of ONH restricted diffusion in AION.

Our study is limited by its retrospective and single-center design. Prospective investigations are needed to validate our findings in extended GCA cohorts. Additionally, technical heterogeneity needs to be considered as DWI-MRI scans were acquired using varying scanners, receive coils and sequence protocols. Our routine DWI-MRI protocols were not optimized to visualize the optic nerve. Nevertheless, good diagnostic accuracy was found for the identification of ION in GCA, which supports the clinical applicability of our findings. Finally, we did not perform ADC-value measurements due to technical limitations^32^, but performed a visual qualitative evaluation of the ADC maps to confirm true diffusion-restriction of the optic nerve in GCA patients. We further acknowledge that the specificity of DWI for ischemic optic nerve changes deserves further study. Rarely, acute inflammatory conditions of the optic nerve or optic nerve neoplasia could yield false positive results when solely considering DWI images^26^. However, differentiation should be readily possible by examining further imaging aspects or clinical and paraclinical findings. Further care must be taken not to misjudge T2-shine-through-associated DWI-hypersignal (e. g. due to inflammation) as true ischemic diffusion restriction by careful consideration of the ADC values.

To summarize, our study finds that standard DWI-MRI is capable of visualizing anterior and posterior ON ischemia in GCA patients with high sensitivity and specificity, as well as substantial inter-rater reliability. DWI may complement ophthalmologic examination in patients with acute loss of vision.

## Data Availability

The datasets generated and/or analyzed during the current study are available in the DRYAD repository (10.5061/dryad.59zw3r2bk).

## References

[1] Nordborg C, Johansson H, Petursdottir V, Nordborg E (2003). The epidemiology of biopsy-positive giant cell arteritis: Special reference to changes in the age of the population. Rheumatology (Oxford).

[2] Crowson CS (2011). The lifetime risk of adult-onset rheumatoid arthritis and other inflammatory autoimmune rheumatic diseases. Arthritis Rheum.

[3] Hayreh SS (2021). Giant cell arteritis: Its ophthalmic manifestations. Indian J. Ophthalmol..

[4] Maz M (2021). American college of rheumatology/vasculitis foundation guideline for the management of giant cell arteritis and takayasu arteritis. Arthritis Care Res. (Hoboken).

[5] Nichani P, Biousse V, Newman NJ, Micieli JA (2021). Vision loss from giant cell arteritis in patients with other ocular diagnoses. J. Neuroophthalmol..

[6] Hayreh SS (2004). Posterior ischaemic optic neuropathy: clinical features, pathogenesis, and management. Eye (Lond).

[7] Ozsunar Y, Sorensen AG (2000). Diffusion- and perfusion-weighted magnetic resonance imaging in human acute ischemic stroke: technical considerations. Top. Magn. Reson. Imaging..

[8] Cauquil C (2012). Diffusion MRI and tensor tractography in ischemic optic neuropathy. Acta Neurol. Belg..

[9] Park JY, Lee IH, Song CJ, Hwang HY (2012). Diffusion MR imaging of postoperative bilateral acute ischemic optic neuropathy. Korean J. Radiol..

[10] Khan AA, Hussain SA, Khan M, Corbett JJ (2012). MRI findings of bilateral posterior ischemic optic neuropathy in postcardiac transplant patient. Neurologist.

[11] Al-Zubidi N, Stevens S, Fung SH, Lee AG (2014). Diffusion-weighted imaging in posterior ischemic optic neuropathy. Can. J. Ophthalmol..

[12] Joos ZP, Adesina OO, Katz BJ (2014). Posterior ischemic optic neuropathy in the setting of posterior reversible encephalopathy syndrome and hypertensive emergency. J. Neuroophthalmol..

[13] Daubler BF, Kamli A, Runge VM (2014). Readout-segmented diffusion-weighted imaging in a critical anatomic area - diagnosing posterior ischemic optic neuropathy (PION). Rofo.

[14] Shams PN, Policeni B, Carter KD, Shriver E, Thurtell MJ (2016). Bilateral septic cavernous sinus thrombosis, congestive orbitopathy, and ischemic optic neuropathy. Can. J. Ophthalmol..

[15] Ragam A, Agemy SA, Dave SB, Khorsandi AS, Banik R (2017). Ipsilateral ophthalmic and cerebral infarctions after cosmetic polylactic acid injection into the forehead. J. Neuroophthalmol..

[16] Distefano AG, Pasol J (2018). Posterior ischemic optic neuropathy after blepharoplasty. J. Neuroophthalmol..

[17] Harrar DB (2018). Diffusion-weighted imaging changes in a child with posterior ischemic optic neuropathy. Pediatr. Neurol..

[18] Aldrees SS, Micieli JA (2020). Rapidly sequential vision loss from posterior ischemic optic neuropathy due to methicillin-susceptible staphylococcus aureus bacteremia. J. Neuroophthalmol..

[19] Kaushik KS, Acharya UV, Krupa L (2022). Dual hit - magnetic resonance imaging in concomitant anterior and posterior ischemic optic neuropathy in a case of rhino-orbital mucormycosis and COVID-19. Indian J. Ophthalmol..

[20] Al-Shafai LS, Mikulis DJ (2006). Diffusion MR imaging in a case of acute ischemic optic neuropathy. AJNR Am. J. Neuroradiol..

[21] Chen JS, Mukherjee P, Dillon WP, Wintermark M (2006). Restricted diffusion in bilateral optic nerves and retinas as an indicator of venous ischemia caused by cavernous sinus thrombophlebitis. AJNR Am. J. Neuroradiol..

[22] Klein JP (2009). Diffusion-weighted magnetic resonance imaging of bilateral simultaneous optic nerve infarctions. Arch. Neurol..

[23] Mathur S, Karimi A, Mafee MF (2007). Acute optic nerve infarction demonstrated by diffusion-weighted imaging in a case of rhinocerebral mucormycosis. AJNR Am. J. Neuroradiol..

[24] Purvin V, Kuzma B (2005). Intraorbital optic nerve signal hyperintensity on magnetic resonance imaging sequences in perioperative hypotensive ischemic optic neuropathy. J. Neuroophthalmol..

[25] Verma A, Jain KK, Mohan S, Phadke RV (2007). Diffusion-weighted MR imaging in posterior ischemic optic neuropathy. AJNR Am. J. Neuroradiol..

[26] Bender B (2014). Diffusion restriction of the optic nerve in patients with acute visual deficit. J. Magn. Reson. Imaging.

[27] Biotti D (2011). Diffusion MR imaging of the optic nerve in a patient with giant-cell arteritis and vertebral artery vasculitis. Eur. Neurol..

[28] Kim DD, Budhram A, Pelz D, MacDonald M (2016). Teaching NeuroImages: Orbital infarction syndrome from giant cell arteritis. Neurology.

[29] Hunder GG (1990). The American college of rheumatology 1990 criteria for the classification of giant cell arteritis. Arthritis Rheum.

[30] Unizony SH (2013). Design of the tocilizumab in giant cell arteritis trial. Int. J. Rheumatol..

[31] Chrysidis S (2018). Definitions and reliability assessment of elementary ultrasound lesions in giant cell arteritis: A study from the OMERACT large vessel vasculitis ultrasound working group. RMD Open.

[32] Danyel LA, Bohner G, Connolly F, Siebert E (2021). Standard diffusion-weighted mri for the diagnosis of central retinal artery occlusion: A case-control study. Clin. Neuroradiol..

[33] Danyel LA (2021). Time course and clinical correlates of retinal diffusion restrictions in acute central retinal artery occlusion. AJNR Am. J. Neuroradiol..

[34] Dejaco C (2018). EULAR recommendations for the use of imaging in large vessel vasculitis in clinical practice. Ann. Rheum. Dis..

[35] Duftner C (2018). Imaging in diagnosis, outcome prediction and monitoring of large vessel vasculitis: A systematic literature review and meta-analysis informing the EULAR recommendations. RMD Open.

[36] Siemonsen S (2015). 3T MRI reveals extra- and intracranial involvement in giant cell arteritis. AJNR Am. J. Neuroradiol..

[37] Serrano Alcala E, GriveIsern E, Diez Borras L, Salaya Diaz JT (2020). Orbital magnetic resonance imaging to unmask giant cell arteritis. Radiologia (Engl Ed).

[38] Pappolla A, Silveira F, Norscini J, Miquelini L, Patrucco L (2019). Bilateral optic perineuritis as initial presentation of giant cell arteritis. Neurologist.

[39] AlShaker S, Shemesh AA, Margolin E (2018). Enhancement of optic nerve sheath in AAION: A case of visual recovery in fulminant GCA. Can. J. Ophthalmol..

[40] D'Souza NM, Morgan ML, Almarzouqi SJ, Lee AG (2016). Magnetic resonance imaging findings in giant cell arteritis. Eye (Lond).

[41] Chen JJ, Kardon RH, Daley TJ, Longmuir RA (2015). Enhancement of the optic nerve sheath and temporal arteries from giant cell arteritis. Can. J. Ophthalmol..

[42] Liu KC, Chesnutt DA (2013). Perineural optic nerve enhancement on magnetic resonance imaging in giant cell arteritis. J. Neuroophthalmol..

[43] Liu TY, Miller NR (2015). Giant cell arteritis presenting as unilateral anterior ischemic optic neuropathy associated with bilateral optic nerve sheath enhancement on magnetic resonance imaging. J. Neuroophthalmol..

[44] Morgenstern KE, Ellis BD, Schochet SS, Linberg JV (2003). Bilateral optic nerve sheath enhancement from giant cell arteritis. J. Rheumatol..

[45] Gospe SM (2021). Magnetic resonance imaging abnormalities of the optic nerve sheath and intracranial internal carotid artery in giant cell arteritis. J. Neuroophthalmol..

[46] Geiger J (2009). Involvement of the ophthalmic artery in giant cell arteritis visualized by 3T MRI. Rheumatology (Oxford).

[47] Sommer NN (2018). Three-dimensional high-resolution black-blood magnetic resonance imaging for detection of arteritic anterior ischemic optic neuropathy in patients with giant cell arteritis. Investig. Radiol..

[48] de Boysson H (2017). Giant cell arteritis-related stroke: A retrospective multicenter case-control study. J. Rheumatol..

[49] Kilani R, Marshall L, Koch S, Fernandez M, Postel E (2013). DWI findings of optic nerve ischemia in the setting of central retinal artery occlusion. J. Neuroimaging.

[50] Danyel LA, Miszczuk M, Villringer K, Bohner G, Siebert E (2021). Retinal diffusion restrictions in acute branch retinal arteriolar occlusion. Sci. Rep..

[51] Lu P (2017). Assessment of nonarteritic anterior ischemic optic neuropathy with intravoxel incoherent motion diffusion-weighted imaging using readout-segmented echo-planar imaging, parallel imaging, and 2D navigator-based reacquisition. J. Magn. Reson. Imaging.

[52] Fang B (2020). Evaluation of acute anterior ischaemic optic neuropathy using diffusion tensor imaging. Clin. Exp. Optom..

[53] Wang MY, Qi PH, Shi DP (2011). Diffusion tensor imaging of the optic nerve in subacute anterior ischemic optic neuropathy at 3T. AJNR Am. J. Neuroradiol..

[54] Hayreh SS, Podhajsky PA, Zimmerman B (1998). Ocular manifestations of giant cell arteritis. Am. J. Ophthalmol..

[55] Remond P (2017). The central bright spot sign: A potential new MR imaging sign for the early diagnosis of anterior ischemic optic neuropathy due to giant cell arteritis. AJNR Am. J. Neuroradiol..

[56] Hayreh SS (2000). Ischaemic optic neuropathy. Indian J. Ophthalmol..

[57] Hayreh SS (1981). Posterior ischemic optic neuropathy. Ophthalmologica.

